# Pilocarpine-Induced *Status Epilepticus* in Rats Involves Ischemic and Excitotoxic Mechanisms

**DOI:** 10.1371/journal.pone.0001105

**Published:** 2007-10-31

**Authors:** Paolo Francesco Fabene, Flavia Merigo, Mirco Galiè, Donatella Benati, Paolo Bernardi, Paolo Farace, Elena Nicolato, Pasquina Marzola, Andrea Sbarbati

**Affiliations:** 1 Section of Anatomy and Histology, Department of Morphological and Biomedical Sciences, University of Verona, Verona, Italy; 2 Center of Experimental Magnetic Resonance Imaging, University of Verona, Verona, Italy; University of Massachusetts Medical School, United States of America

## Abstract

The neuron loss characteristic of hippocampal sclerosis in temporal lobe epilepsy patients is thought to be the result of excitotoxic, rather than ischemic, injury. In this study, we assessed changes in vascular structure, gene expression, and the time course of neuronal degeneration in the cerebral cortex during the acute period after onset of pilocarpine-induced *status epilepticus* (SE). Immediately after 2 hr SE, the subgranular layers of somatosensory cortex exhibited a reduced vascular perfusion indicative of ischemia, whereas the immediately adjacent supragranular layers exhibited increased perfusion. Subgranular layers exhibited necrotic pathology, whereas the supergranular layers were characterized by a delayed (24 h after SE) degeneration apparently *via* programmed cell death. These results indicate that both excitotoxic and ischemic injuries occur during pilocarpine-induced SE. Both of these degenerative pathways, as well as the widespread and severe brain damage observed, should be considered when animal model-based data are compared to human pathology.

## Introduction

Ischemic and excitotoxic injuries involve a variety of mechanisms that produce different types of neuronal death [1]. Although seizure-induced neuronal injury was originally called “ischemic injury”, pioneering studies by Meldrum and colleagues demonstrated that when ischemia was prevented, seizure activity still produced irreversible neuronal injury [2]. Subsequent studies demonstrated that seizure activity itself can be neurotoxic, and that the primary mechanisms involve excitatory amino acid receptor mediation of excitotoxic insults [3]–[6]. However, recent studies have suggested that prolonged *status epilepticus* (SE) in rats, whether induced by pilocarpine or kainate, also involves profound vascular changes that cause ischemic, as well as excitotoxic injury [7], [8]. Thus, precisely which pathological changes observed after prolonged SE in animals are ischemic in nature, and which are excitotoxic, is unclear. This is an important issue to resolve because prolonged SE is commonly used to induce a chronic epileptic state, and it is generally assumed that all of the SE-induced changes in animals are excitotoxic in nature [9], and closely related to the pattern of cell death exhibited in human temporal lobe epilepsy [2]. The possibility that prolonged SE also involves ischemic injury has significant implications for the interpretation of results generated by pilocarpine-induced epilepsy.

We recently showed that pilocarpine-induced SE produced significant vascular/ischemic effects in the cingulate and somatosensory cortices of Wistar rats [7]. Given the profound vascular effects subsequently reported in the hippocampi of pilocarpine- and kainate-treated rats [8], prolonged SE may be an insult more severe than that observed in other experimental epilepsy models, or in humans who exhibit a highly selective and relatively limited pattern of neuron loss [2]. In our previous study, we reported that vascular alterations, characterized by hyperemia and degenerating neurons, were evident in the supragranular layers of the somatosensory cortex and in the layers II/III of the cingulate cortex of Wistar rats 12 h after SE lasting 4 h, whereas immediately adjacent subgranular layers exhibited edema and necrotic cell death [7]. These results suggested that cortical injury caused by pilocarpine-induced SE might involve different mechanisms in adjacent structures because of differences in blood vessels organization and regulation between the two areas. In the present study, we addressed anoxic/hypoxic/ischemic alterations and excitotoxic injury in the immediate post-SE period (2 h post-SE) to evaluate vascular changes, neurodegenerative patterns, and possible differences in the expression of genes that may mediate different mechanisms of SE-induced neuronal death. In this study, we used a multidisciplinary approach, based both on *in vivo* (MRI) and ex vivo (immunohistochemestry, electron microscopy, superarrays) analysis.

## Materials and Methods

### Animals

**Figure 1:**
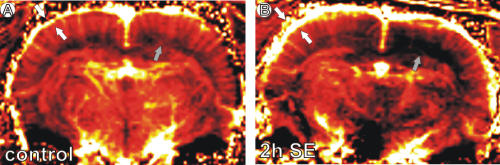
Compared to controls (A), pilocarpine-treated animals 2 h post-SE onset (B) showed a specific laminar pattern in rCBV distribution, with increased values in the more superficial layers (white arrows) paralleled by a decrease in rCBV values in the deeper layers, indicating an ischemic area (gray arrows).

Male adult Wistar rats (80–90 days of age), were kept under controlled environmental parameters and veterinarian control (Fig. 1). The animals were habituated to the experimenters for at least two weeks prior to the procedures employed in the present study. The experiments received authorization from the Italian Ministry of Health, and were conducted following the principles of the NIH Guide for the Use and Care of Laboratory Animals, and the European Community Council (86/609/EEC) directive. All efforts were made to minimize the number of animals used and avoid their suffering. Seizures were induced by pilocarpine injections as reported in previous studies [7]. Briefly, to minimize the peripheral effects of pilocarpine, the cholinergic antagonist methyl-scopolamine (1 mg/kg, s.c.) was administered to all animals. After 30 min the rats were randomly divided in two groups; those of the first group were injected i.p. with pilocarpine (380 mg/kg; Sigma Chemical Co., St. Louis, MO) diluted in 0.01 M phosphate-buffered saline, pH 7.4 (PBS), whereas animals of the control group were injected with PBS. Only animals who reached stage 5 of the Racine's scale [10] and experienced a 2 h SE were included in the epileptic group examined at 2 h and 24 h. In order to standardize the different experimental groups, all animals (control, 2 h and 24 h after SE-onset, respectively) were treated by diazepam administration (Sigma, one i.m. injection, at the dose of 4 mg/kg) 2 h after pilo (or saline) injection. Two hours after SE onset, four animals per group were decapitated and brains dissected out from the skull for superarray analysis, whereas eight more animals per (group in the same experimental conditions) were perfused by a low­viscosity resin for vascular cast technique (n = 4) or 4% para-formaldehyde (n = 4) for EM analysis. Ten animals per group were processed by MRI scanning at the same time-point (2 h after SE-onset), and immediately after perfused for histological procedures. Follow-up at 24 h after SE-onset was performed only with the techniques not used in the previous experiment [7].

### MRI analysis

MRI technique has been widely used in studies of experimental epilepsy [11]. MRI experiments were performed using a Bruker Biospec Tomograph equipped with an Oxford, 33-cm-bore, horizontal magnet operating at 4.7 T. Animals were anesthetized with 1% halothane in 1 L of oxygen in air per minute (initial dose: 4% halothane); rectal temperature and heart rate were monitored and were similar in control and post-SE animals. The rats were placed into a 7.2-cm-i.d. bird cage transmitter coil. The signal was received through a 2-cm surface coil, actively decoupled from the transmitter coil, and placed directly on the animal's head. Three mutually perpendicular slices were acquired through the brain as scout images. Five contiguous 2-mm-thick slices were imaged starting 1 mm posterior to the olfactory bulbs using a RARE sequence with TEoff = 65.2, FOV = 4×4 cm^2^, matrix size: 256×128, zero-filled at 256×256.

### rCBV

In this experiment an USPIO particle (Sinerem®, kindly supplied by Guerbet, Aulnay-Sous-Bois, France) was used as a contrast agent. Sinerem® is constituted by an iron-oxide core of about 6 nm diameter coated by dextran (coated particle dimensions of about 20 nm) and is characterized by a blood half-time longer than 2 h in rats [12]. Sinerem® (6 mg iron/kg) was dissolved in saline and injected in the tail vein.

rCBV acquisition protocols and image analysis techniques were as described previously [7], [13]. Briefly, transversal multislice gradient echo images were acquired before and two minutes after administration of Sinerem® with the following parameters: TR = 350 ms, TE = 15 ms, flip angle = 30°, Field of view 4×4 cm^2^, matrix size 256×256, slice thickness = 2 mm. Five continuous slices were acquired.

### rTTP maps

First passage images were acquired during the arrival of the contrast agent in the brain. A snapshot flash sequence was used, with TR = 27.9 ms, TE = 10 ms, flip angle = 7°, matrix size 128×32 (zero filled at 128×128). A single slice 2mm thickness was acquired. The time interval among images was 0.2 sec: three images were acquired before and 17 images after the bolus administration of Sinerem.

The dynamic data were then analyzed to calculate the peak enhancement and especially the relative TTP. The latter parameter is related to mean transit time and blood flow. TTP was evaluated on the signal intensity plot as the time point of maximal signal reduction. Relative TTP was obtained as a difference between TTP and time of arrival TA: rTTP = TTP−TA, where the time of arrival is the time for contrast material to arrive in the brain [14].

Peak enhancements were calculated by the relation: (SImin-SIpre)/SIpre, where SImin is the signal intensity at the time of maximal signal drop and SIpre is the average signal intensity of three points before contrast administration.

### Histochemical analysis

Free floating sections were washed in PBS at room temperature and permeabilized for 1 hour in PBS containing 0,3% Triton X-100, 1% bovine serum albumin and 2% normal goat serum, the same solution was used to dilute the antibodies. Subsequently, sections were incubated overnight in a mixture of rabbit polyclonal anti-Agrin with mouse monoclonal anti-Glial Fibrillary Acidic Protein (GFAP; Cymbus Biotechnology LTD, Chemicon International, CA, USA). After washes, sections were incubated in fluorescein (FITC) conjugated affinity purified goat anti-rabbit IgG (1∶200; Jackson Laboratories, INC; Baltimore, PA) and Cy3-conjugated affinity purified goat anti-mouse IgG (1∶200; Jackson Laboratories), for two hours at room temperature. Sections were collected on polylysine-coated slides, mounted with N-propyl gallate and viewed with a Zeiss LSM 510 confocal microscope equipped with argon (488 nm) and helium/neon (543 nm) excitation beams. Sequential acquisition, i.e., one color at a time, was utilized on double-label tissues to avoid side-band excitation of the other fluorophore. Sections treated as above, but in the absence of the primary or secondary antibody, were used as control. To label injured neurons, the Dark Neuron stain method was used as described by others [15].

### Transmission Electron Microscopy

For ultrastructural analysis, dissected tissue blocks from 16 animals (4 animals per group) were fixed by immersion in 2% glutaraldehyde in 0.1 M phosphate buffer (PB), pH 7.4, for 2 hours at 4°C. After rinsing in 0.1 M PB, the specimens were postfixed in 1% OsO4 in PB for 1 hour, dehydrated in graded concentrations of acetone and embedded in a mixture of Epon and Araldite (Electron Microscopic Sciences, Fort Washington, PA, USA). Semithin sections, at 1 µm thickness, were stained with toluidine blue. Ultrathin sections were cut at 70 nm thickness on an Ultracut-E ultramicrotome (Reichert-Jung), stained with lead citrate and uranyl acetate and observed on a Zeiss EM 10 electron microscope (Zeiss, Oberkochen, Germany).

### Vascular Casts

Vascular casts were obtained as described in our previous work [16]. For the vascular cast preparation, two rats per group were anesthetized with sodium pentobarbital and thoracotomized. A catheter was introduced into the arch of the aorta through the left ventricle and the right atrium was opened. The circulatory system was rinsed with phosphate-buffered saline and a freshly prepared solution of a low­viscosity resin, Mercox CL-2B (Dainippon Ink & Chemicals, Tokyo, Japan) was injected along the same route. Maceration of the brain tissue was performed using a 10% solution of potassium hydroxide until only the resin casts of blood vessels remained. The specimens were rinsed by several passages of distilled water, and then frozen. The casts were freeze-dried (Modulyo, Edwards-Kniese, Marburg, Germany), fixed to stubs with colloidal silver, sputter-coated with gold (MED 010, Balzers), and examined under a SEM (DSM 690, Zeiss).

### MicroArrays

mRNA was isolated from the cingulate cortex of 4 rats in each group. Tissue was selected in supragranular and subgranular layers. The mRNA was used as the template for generating a cDNA library. cRNA labeled with dUDP-biotin (Enzo Roche Molecular Biochemicals, Mannhein, Germany) has been retro-transcripted. dUDP-biotin-cRNA was purified by and hybridizated on different OligoGEArrays containing probes specific for genes implicated in signal transduction pathways (95 genes, 2 blanks, 6 negative controls, 9 positive controls) (Oligo GEArray Rat Apoptosis Microarray ORN-014, SuperArray Bioscience Corporation), apoptosis (96 genes, 1 blanks, 6 negative controls, 9 positive controls) (Oligo GEArray Rat Apoptosis Microarray ORN-012, SuperArray Bioscience Corporation) and DNA-damage response (113 genes, 2 blanks, 4 negative controls, 9 positive controls) (Oligo GEArray Rat Apoptosis Microarray ORN-029, SuperArray Bioscience Corporation). The hybridization pattern was revealed by CDP-Star® substrate fluorescence using Chemiluminescent Detection Kit and recorded on a X-ray film.

All the steps of the procedure were performed using reagent kits purchased by SuperArray® Bioscience Corporation (Frederick, MD, USA) and closely following manufacturer's instructions.

The film was acquired using a desktop scanner and saved as a grayscale TIFF file. Data from array were analyzed using GEArray Analyzer software (Superarray Bioscience Corporation).

### Statistical evaluation

For MRI data analysis, the difference between T2W and rCBV values obtained in control vs pilocarpine-treated rats was evaluated with one-way analysis of variance (ANOVA), followed by the LSD post-hoc test, setting the significance at p<0.05. The same statistical approach was used for immunohistochemical and vascular cast analysis.

For microarrays analysis, genes with values <0.20 were considered to be “not expressed”. Differences of expression of 1.5-fold or more between the lineages under analysis were considered significant. A cross-validation between different arrays is provided.

## Results

### Cerebral blood volume and rTTP

Compared to controls, animals pilocarpine-treated showed a specific laminar pattern in rCBV distribution, with increased values in the more superficial layers (control: 0.32±0.13 a.u.; epileptic: 0.63±0.16 a.u.; p<.0001) and decreased in rCBV values in the deeper layers (control: 0.21±0.07 a.u.; epileptic: 0.11±0.05 a.u.; p<.0001). The decreased rCBV values indicate an ischemic area (Fig. 1).

**Figure 2:**
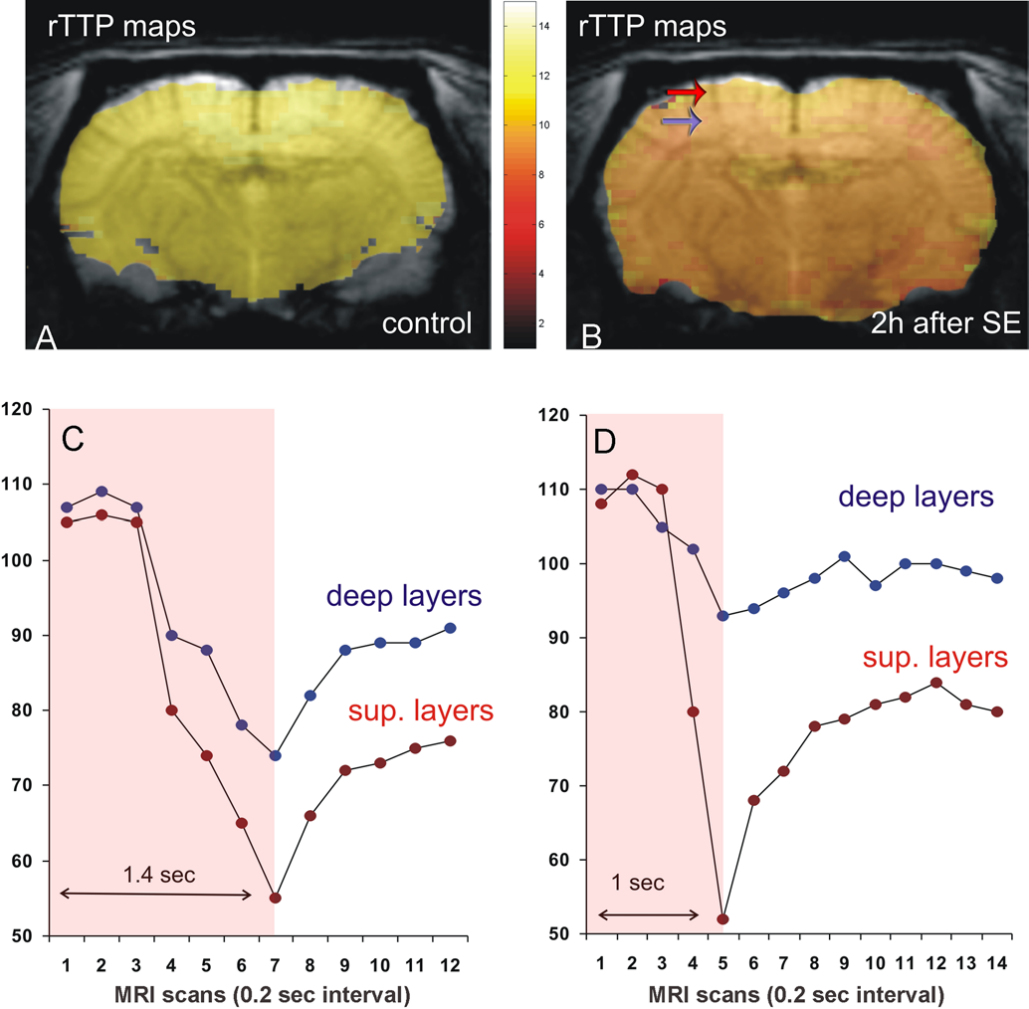
Peak enhancement in control (A, C) and pilocarpine-treated animals (B, D). Compared to controls, in rats during SE, sub-granular layers showed a decreased contrast medium peak concentration (D, blue dots), indicating a relative ischemic core, whereas supra-granular layers were characterized by hyperemia (D, red dots). Overlay rTTP maps on source images showing a generalized increase in blood flow rate in pilocarpine-treated (B) versus control brain (A). These alterations in the cerebral cortex of pilocarpine-treated rats present a specific spatial distribution (supra- (red arrow) versus sub-granular layers (blue arrow)).

rTTP was used as a surrogate measure of mean transit time [14], to evaluate blood flow alterations in epileptic vs control brains. rTTP maps show a generalized increase (about 0.4 secs) in blood flow rate in epileptic vs control brains, with a spatially distinct pattern (Fig. 2). Maximal signal drop values, obtained by first passage technique, resemble those obtained by images acquired at the equilibrium distribution of USPIO (all rCBV maps reported in Fig. 2).

### Vascular cast

**Figure 3:**
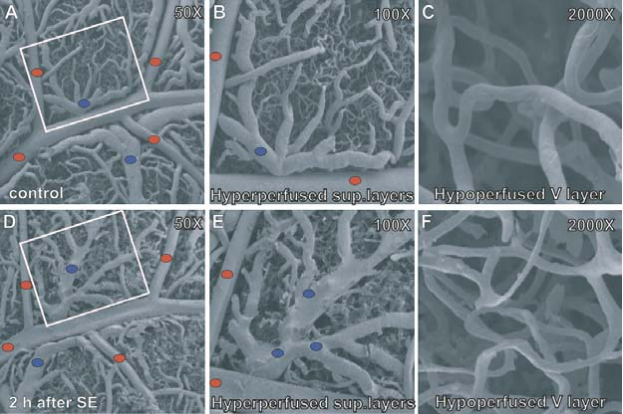
Vascular casts revealed structural alterations in brain vessels 2 h after SE-onset. In the superficial, hyperperfused zone, veins were increased in diameter (B, E) whereas in the hypoperfused, edematous subgranular region, veins appeared collapsed (C, F).

Vascular changes were evident in animals injected by pilocarpine versus controls, both in the principal vessels (Fig. 3A–B and D–E) and in the microcirculation (vessels diameter <100 µm, Fig. 3C, F). Principal arteries and veins were flattened and veins were 20% larger in diameter than in control animals (diameter: control: 149.03 µm±4.5; exp: 207.54 µm±6.1; p<.02). Microvessels were clearly altered in the deeper layers where flattened vessels were observed (control: 7.67 µm±0.9; exp: 3.51 µm±0.2; p<.0001).

### Endothelial permeability, reactive gliosis and neuronal death

**Figure 4:**
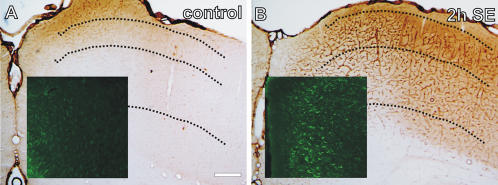
Agrin expression in control (A) and pilocarpine-treated (B) animals. Increased agrin expression in the endothelial cells is evident in both superficial and deeper layers 2 h after SE-onset. This increase was more evident in the supergranular than in the subgranular layers. Localization of GFAP-like immunoreactivity revealed a selective increase in astrocytic GFAP expression in the less acutely damaged superficial layers (square).

Agrin expression was dramatically increased 2 h after SE in the Wistar rat cerebral cortex (Fig. 4; densitometric evaluation: control: 2.13±0.2 a.u.; 2 h: 5.27±1.1 a.u.; p<.001). This increase was more evident in the supergranular than in the subgranular layers (Fig. 4). Reactive astrocytosis paralleled agrin expression (Fig. 4; densitometric evaluation: control: 4.22±0.9 a.u.; exp: 6.56±1.7 a.u.; p<.001), as well as dark neuron staining (cell number/41,400 µm^2^ evaluation: supragranular layers: control: 0±0; 2 h: 0±0; 24 h: 3.2±1.4. Subgranular layers: control: 0±0; 2 h: 12.2±2.8; 24 h: 68.1±5.3; p<.0001).

### Ultrastructural analysis

**Figure 5:**
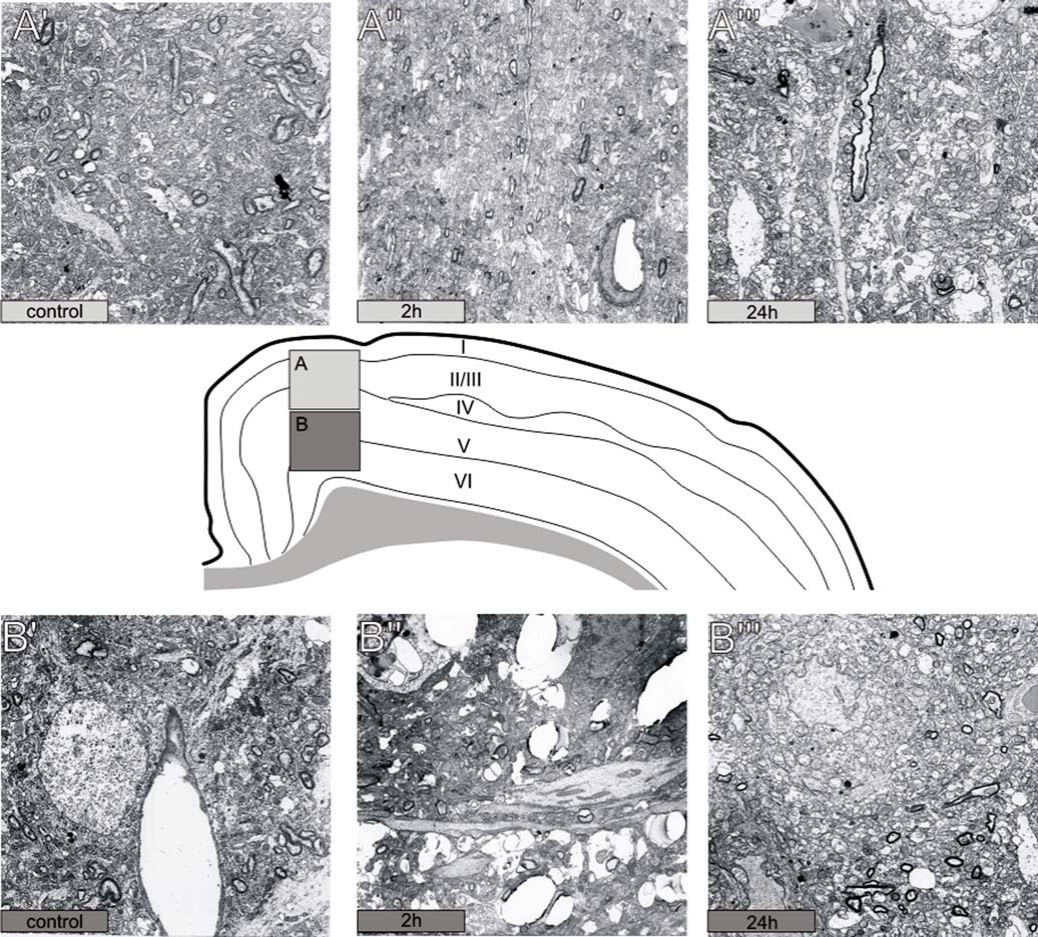
EM images in control animals and 2 h and 24 h after SE in both supergranular (A, A′, A″) and subgranular levels (B, B′, B″). Subgranular layers are characterized of profound tissue damage 2 h after SE (B′) whereas supergranular layers at this time point appear to be normal except for the perivascular edema (A′). Twenty-four h after SE pathological evidences are detectable in both areas (A″′, B″′).

The ultrastructural analysis demonstrated the histological and cytological alterations occurring after pilocarpine-induced SE (Fig. 5). Two hours after SE supragranular layers appeared almost normal, with only a few morphological indicators of histopathology (i.e.: the perivascular edema in Fig. 5A″), whereas subgranular layers at this early time-point exhibited a severe parenchymal changes and vacuolisation (Fig. 5B″). This pattern were changed 24 h post-SE, when histopathological alterations were apparent in both the superficial (Fig. 5A″′) and inferior (Fig. 5B″′) layers.

### Gene arrays analysis

Gene array data were analyzed comparing mRNA expression of 2 h versus control and 24 h versus 2 h. We cross-validated gene array by the mean of correlation index of 12 genes present in two or more different arrays (signal transduction vs apoptosis: r = 0.956; apoptosis vs DNA-damage: r = 0.986; signal transduction vs DNA-damage: r = 0.999).

In the subgranular layers, *Bcl2a1, Bcl10* expression was upregulated and *Bax* expression was downregulated. DNA-repairing (upregulation of *PCNA, Xrcc1, Xrcc3*), was noted 2 h after SE (Table 1). This activation was paralleled by neuroprotective mechanisms (upregulation of *NGF*) (Table 1). In these early stages of cortical alteration, in the supragranular layers a different and more complex gene-expression pattern was evident. Signals of cellular stress (upregulation of *Hexonkinase2* and *Ubiquitin conjugating enzymes*), paralleled by an downregulation of *heat shock protein 27* were observed in these layers at 24 h compared to 2 h after SE levels (Table 1).

**Table 1:** MicroArray analysis of cortical sample from supragranular vs subgranular layers at different time-points.

	2 h *versus* control		24 h *versus* 2 h	
	**Upregulation**		**Upregulation**	
Supragranular layers (II/III)	c-*fos* (9.6)	FBJ murine osteosarcoma viral oncogene homolog	Apex 1 (689,5)	Apurinic/apyrimidinic endonuclease 1
	Ube 2d3 (2.85)	Ubiquitin-conjugating enzyme E2D 3	Bax (2.0) (2.54)	Bcl-2associated X protein
	Ube 2i (2.0)	Ubiquitin-conjugating enzyme E2I	Cib 1 (86.8)	Calcium and integrin binding protein 1
	TNF R sf1a (5.25)	Tumor necrosis factor receptor 1a	Becn1 (3.74)	Beclin 1
	Cdkn2a (33.7)	Cyclin dependent kinase inhibitor 2a	Ube2i (2.7)	Ubiquitin-cojugating enzyme E2I
	Cdk 2 (2.1)	Cyclin dependent kinase 2	Hspb1 (2.9)	Heat Shock 27 KDa Protein
	Hk2 (5.5)	Hexokinase 2	Ctsd (2.5)	Cathepsin D
	Igf bp3 (2.09)	Insulin-like growth factor binding protein 3	Fn1 (5.2)	Fibronectin 1
			IL-4 R (2.2)	Interleukin 4 receptor
			Ccl2 (7.5)	Chemokine (C-C motif)ligand 2
			a2m (9.0)	Alpha 2 microglobulin
			bmp4 (5.7)	Bone morphogenetic protein 4
			Igfbp3 (2.26)	Insulin-like growth fact. bin prot 3
			Csn2 (4.6)	Casein beta
			Cdk inhib 1b (4.7)	Cyclin dependent kinase inhibitor 1B
			PCNA (3.09)	Proliferatine cell nuclear antigen
			Ddit3 (2.97)	DNA-damage inducile transcript 3
			Smc11 (4.75)	Structural maintenance chromosomes
			Ercc1 (5.8)	Similar to excision repair
			Mre11a (3.0)	Meiotic recombination 11 homolog A
			Mgmt (2.0)	O-6-metylguanine-DNA methyltransferase
			Trex1 (4.8)	Similar to 3-5 exonuclease
			Ercc3 (11.4)	Similar to TFII basal transcription factor complex helicase XPB subunit
			Tdg (13.8)	Thymidine-DNA glycosilase
			Atf2 (4.6)	Activating transcription factor 2
			Rad 23 a (0.5)	Similar to UV excision repair protein RAD23 homolog A
			Pnkp (4.56)	Similar to polynucleotide kinase 3-phosphate
	**Downregulation**		**Downregulation**	
	Hspb1 (0.48)	Heat shock 27 KDa protein	TNFsf13 (0.59)	Tumor necrosis factor ligand 13
	Bax (0.36)	Bcl-2associated X protein	Birc1 (0.25)	Baculoviral IAP repeat-containing 1b
	Csf2 (0.48)	Colony stimulating factor 2	C-fos (0.236)	FBJ murine osteosarcoma viral oncogene homolog
	Bmp4 (0.288)	Bone morphogenetic rotein 4	Ei24 (0.42)	Similar to EI24
	Csn2 (0.418)	Casein beta		
	Rad 23(0.22)	Similar to excision repair protein RAD23 homolog A		
	IL-2 (0.26)	Interleukin 2		
	Hoxb1 (0.160)			
	**Upregulation**		**Upregulation**	
Subgranular layers (V/VI)	c-*fos* (9.6)	Early growth response 1	Ercc1 (2.0)	Similar to excision repair cross-complementing rodent repair deficiency, compl.group1
	V-Jun (3.7)		Rad23a (3.6)	Similar to UV excision repair protein RAD23 homolog A
	NGF (2.0)	Nerve growth factor	Rad23b (8.0)	Similar to UV excision repair protein RAD23 homolog A
	TNFR1a (2.9)	Tumor necrosis factor receptor 1a	Cspg6 (7.45)	Caspase 6
	Bcl 10 (16.4)	B-cell leukemia/lymphoma 10	Cib 1 (4.8)	Calcium and integrin binding protein 1
	Bcl 2 a 1 (5.6)	B-cell leucemia/lymphoma 2 related protein A1	Dclre1a (4.5)	Similar to SNM1 protein
	PCNA (2.1)	Proliferatine cell nuclear antigen	Atrx (7.9)	Alpha thalassemia/mental retardation sindrome X-linked homolog (homolog)
	Xrcc1 (2.29)	x-ray repair complementing defective repair	Ddit 3 (7.7)	DNA-damage inducile transcript 3
	Xrcc3 (2.29)	Similar to DNA-repair protein XRCC3	Odc1 (3.8)	Ornithine decarboxylase 1
			Csf2 (5.7)	Colony stimulating factor 2
			NGF (1.7)	Nerve growth factor
			Bnip 3 (2.9)	BCL2/adenovirus E1B 19 KDa-interacting protein 1
			TNF R sf 1 a (2.4)	Tumor necrosis factor receptor 1A
			DAP 3 (2.9)	ESTs, moderately similar to death associated protein 3
			Myd 88 (3.67)	Myeloid differentiation primary response gene 88
			Bcl2a1 (4.8)	B-cell leucemia/limphoma 2 related protein A1
			Bnip 1 (9.47)	BCL2/adenovirus E1B 19 kDa-interacting protein 1
			Becn 1 (2.9)	Beclin 1
			Hspb1 (3.0)	Heat shock 27 KDa protein
	**Downregulation**		**Downregulation**	
	Bax (0.27)	Bcl-2associated X protein	c-*fos*	FBJ murine osteosarcoma viral oncogene homolog
	Bmp4 (0.13)	Bone morphogenetic protein 4	Prkeb1 (0.43)	Protein kinase c beta 1
	Egfr (0.3)	Epidhermal growth factor receptor	Cdkn2a (0.07)	Cyclin dependent kinase inhibitor 2°
	Bid3 (0.33)	BH3 interacting domain, apoptosis agonist	Bcl 10 (0.14)	B-cell CLL/lymphoma 10
	TNFsf10 (79)	Tumor necrosis factor ligand 10		

Twenty-four h after SE, in the subgranular layers, several genes involved in the DNA-repairing processes (*Ercc1, Rad23a, Rad23b, Ddit3, Atrx, Mre11a, Mgmt, Tdg, Rad 23a*), as well as neuroprotective (*NGF*) and anti-apoptotic genes (*Bcl2, Birc7, Bnip3, Becn1*) were upregulated (Table 1). Conversely, in the supragranular layers, a more composite pattern was found. Evidences of apoptotic cell death, mediated by an upregulation of *Bax* and a downregulation of *Birc1,* as well as DNA-repairing genes (*Ercc1, Trex1, Ercc3, Ddit3, PCNA*) and inflammatory processes (*IL-4R, Chemokine ligand 2*) were noted (Table 1) 24 h after SE. An indication of protein-degradation alteration (upregulation of *Catepsin D, Hsp27, Ubiquitin conjugating enzymes*) was also evident (Table 1).

## Discussion

The results of this study indicate that a two different patterns of vascular and neurodegenerative phenomena can be identified in the Wistar rat neocortex after pilocarpine-induced SE. First, in the subgranular layers (cortical layer V and VI), 2 h after SE-onset, ischemic (anoxic) alterations are followed by necrotic cell death and edema. Second, the supragranular area (cortical layers II/III), is characterized by hyperperfusion and delayed apoptotic cell death. Thus, prolonged SE-induced brain damage appears to involve both ischemic and excitotoxic mechanisms.

### Vascular alterations

Cerebral blood flow and cerebral metabolic rate alterations during prolonged epileptic seizures in rats were extensively studied in the 70's [17], [18]less frequently recently [19], [20]. In early studies, a marked increase in CBF substantially exceeding the increase in cerebral oxygen consumption, was noted within a few seconds of the onset of a generalized epileptic seizure and lasted for several hours [18]. In this study, we report for the first time that vascular alterations are not homogeneous in the cerebral cortex after 2 h pilocarpine-induced SE. Vascular cast and MRI observations of superficial arteries and veins in the supragranular layers indicate increased blood volume, whereas in the deeper layers of the cortex (subgranular layers), microcirculation studies showed a reduced diameter, resulting in an ischemic core. Furthermore, our TTP study confirmed the CBF alterations previously reported [17], demonstrating a specific spatial distribution (supra- versus sub-granular layers). Histopathological analysis, based on Nissl staining and agrin detection strongly suggest the vascular alteration and endothelial changes due to pilocarpine-induced SE. In facts, agrin is supposed to mediated the formation and maintenance of cerebral microvascular impermeability [21]. As a component of the brain microvascular basal lamina, agrin may impact pathological processes in which the vascular permeability barrier is defective [21]. The correlation between vascular alterations and neurodegeneration have been obtained by dark neuron (DN) staining, a silver-impregnation techniques used to label the cell body and dendritic processes of degenerating neurons. It has long been recognized that many dying and dead neurons, seen in a variety of diseases, exhibit an increased affinity for various silver stains (argyrophilia) [22]. This technique has been used in comparison with the anionic fluorochrome Fluoro Jade B (FJB) in pilocarpine-treated rats [23]; a significant increased percentage of silver-stained profiles at earlier time points (<24 h) compared with Fluoro-Jade positive cells stained at the same time in adjacent brain sections was reported [23].

### Anoxic/hypoxic damage and neurodegeneration

Our results indicate that SE-induced neurodegeneration in the cerebral cortex of Wistar rats involves vascular insult, that presumably results in an alteration of the intra- and extra-cellular ionic homeostasis due to release of excitotoxic aminoacids (e.g: glutamate) in the extracellular compartment, with a consequent cellular integrity loss and the presence of initial necrotic phases [24]. One of the most common consequences of ischemic insult is oxygen free radical-accumulation, which causes extensive damage [24], [25]. In the ischemic area, necrotic mechanisms are frequently detected as well as expression of anti-excitotoxic, anti-inflammatory and anti-apoptotic mechanisms [24]. The efficacy of such protective mechanisms may be dependent on the severity of the insult.

Our results indicate that the subgranular layers 2 h after SE are morphologically characterized by a massive necrotic degeneration and tissue alteration, while the transcriptional profile reveals an activation of anti-apoptotic (Bcl2 and *Bcl10* up-regulation paralleled by a downregulation of *Bax*), neuroregenerative (*NGF* and *egr-1* upregulation) and DNA-reparative (*PCNA, Xrcc1* and *Xrcc3* upregulation) pattern. Members of the Bcl2 family are thought to be proteins involved in regulation of neurodegenerative phenomena. The expression of Bcl2 has been found to inhibit apoptosis after various types of neuronal injuries [26], as well as to inhibit necrosis [27]. In order to inhibit apoptosis, Bcl2 forms a heterodimer with Bax [28]. Bax can also dimerise with itself, and appears to induce apoptosis when overproduced [29]. It has been proposed that the balance of Bax/Bcl2 in a cell is a critical factor determining whether a cell will undergo apoptosis [30].

Several studies reported a neuroprotective and reparative role of NGF in neurodegenerative diseases [31]. In particular, the increased production of NGF and other trophic factors in CNS during neurodegenerative disease may suppress inflammation by switching the immune response to an anti-inflammatory, suppressive mode [32].

Ischemia-related DNA alterations have been extensively reported [24]; activation of DNA-repairing mechanisms (e.g.: *Xrcc* family) has been considered as an extreme response also in other experimental model of epilepsy [33].

### Excitotoxic cell death

The supragranular layers exhibited a delayed programmed cell death temporally and spatially distinct from the fast neuronal and glial cell death (pannecrosis, minute to hours) in the deeper layers [34]. In fact, 24 h after SE, the supragranular layers exhibited an upregulation of *Bax*, suggesting a pro-apoptotic switch. We also found an upregulation of DNA-repairing enzymes (for example, *PCNA, Ercc1, Ercc3, Trex1*) and neurodegeneration-associated proteins (e.g.: *fibronectin1*, *Igf*).

The neuroprotective molecule *Hsp 27* is constitutively expressed in mammalian rat brain [35] and is upregulated after ischemic insults [36] or lesions [37]. Interestingly, Plumier and colleagues [38] demonstrated that this gene is increased 2.5 fold after kainic acid-induced SE. In agreement with these data, we have found a moderate expression of *Hsp27* both in supragranular and subgranular layers of control animals, with a dramatic upregulation 24 h after SE [7], probably as a response of the brain against neurodegeneration and misfolded protein accumulation.

### Summary

Taken together, the data obtained in this study, point out that a) the cerebral cortex responds in different ways to pilocarpine stimulation, with ischemic, necrotic processes in the subgranular layers and presumably excitotoxic, apoptotic cell death in the supragranular layers; b) these two degenerative pathways take place at different time points, with a delayed degeneration in the supragranular layers. Furthermore, the present findings, pointing out the different pathways involved in the degenerative alterations following SE and the different time-window of the occurrence of such mechanisms, could prompt future studies for putative treatments that can prevent or limiting epileptogenic changes following SE or repeated seizures.
